# Robust Observation Detection for Single Object Tracking: Deterministic and Probabilistic Patch-Based Approaches

**DOI:** 10.3390/s121115638

**Published:** 2012-11-12

**Authors:** Mohd Asyraf Zulkifley, David Rawlinson, Bill Moran

**Affiliations:** 1 Department of Electrical, Electronic and Systems Engineering, Faculty of Engineering and Built Environment, Universiti Kebangsaan Malaysia, 43600 Bangi, Malaysia; 2 Department of Electrical and Electronic Engineering, The University of Melbourne, VIC 3010, Australia; E-Mails: davidjr@unimelb.edu.au (D.R.); wmoran@unimelb.edu.au (B.M.)

**Keywords:** tracking observation, Neyman-Pearson method, Poisson modelling, maximum correlation, histogram intersection, patch matching

## Abstract

In video analytics, robust observation detection is very important as the content of the videos varies a lot, especially for tracking implementation. Contrary to the image processing field, the problems of blurring, moderate deformation, low illumination surroundings, illumination change and homogenous texture are normally encountered in video analytics. Patch-Based Observation Detection (PBOD) is developed to improve detection robustness to complex scenes by fusing both feature- and template-based recognition methods. While we believe that feature-based detectors are more distinctive, however, for finding the matching between the frames are best achieved by a collection of points as in template-based detectors. Two methods of PBOD—the deterministic and probabilistic approaches—have been tested to find the best mode of detection. Both algorithms start by building comparison vectors at each detected points of interest. The vectors are matched to build candidate patches based on their respective coordination. For the deterministic method, patch matching is done in 2-level test where threshold-based position and size smoothing are applied to the patch with the highest correlation value. For the second approach, patch matching is done probabilistically by modelling the histograms of the patches by Poisson distributions for both RGB and HSV colour models. Then, maximum likelihood is applied for position smoothing while a Bayesian approach is applied for size smoothing. The result showed that probabilistic PBOD outperforms the deterministic approach with average distance error of 10.03% compared with 21.03%. This algorithm is best implemented as a complement to other simpler detection methods due to heavy processing requirement.

## 1. Introduction

Obtaining the correct observation for track maintenance is a very challenging task. Tracking accuracy is highly dependent on accurate observation. Improving the accuracy of observation detection and association are two crucial factors in building good trackers, especially in people counting and behaviour analysis systems. Even for a global positioning system [1], observation data from several satellites are optimized in order to provide the best possible coordinate. During complex situations such as illumination changes, clutter and occlusion, robust observations are rarely obtained, which lead most trackers [2,3] to utilize prediction information only. Moreover, null observation and false association sometimes occur, which will hinder tracker performance. The challenge of detecting the tracked object throughout the video is more difficult compared with detecting an object in a database (image processing) due to the non-rigid nature of the object where its appearance varies with time. Even though there is a strong correlation between the current and previous frame in detecting the moving object, the template itself is not perfect, which makes comparison between frames very difficult. The dynamic change between frames is the reason behind the difficulty in establishing and retaining a good set of template. This paper is dedicated to robust observation detection, especially in various challenging environments and surroundings. The processing requirement of the algorithm is quite heavy, so we suggest the user implement a simpler detection algorithm as the primary method and only switch to our algorithm for challenging situations and surroundings. An example of a simple switching mechanism is if the main method fails to detect the observation, *i.e.*, there is no observation detected for that particular scan, then our algorithm is activated to obtain the measurement. We limit this paper to single object tracking only, which is a part of Zulkifley's PhD thesis [4].

Generally, there are two major approaches to obtain the measurement input, either by detection with recognition or detection without recognition. Recognition in this case means we know in the first place to which track a particular observation belongs. Foreground segmentation and optical flow are two methods of obtaining measurement input without recognizing the tracked object. Those algorithms work by detecting moving pixels without knowing the identity of the tracked object, and the detected foreground blob such as from [5] may contain more than a single tracked object. The detected foreground blob must be associated with a specific track in order to know the identity of the object. Multiple hypothesis approach [6] can be employed to optimize the association process so that no merge and split cases are considered. On the other hand, observations can be obtained by recognizing the object using feature-based and template-based recognition. We have pre-stored data of the tracked object where we will find it in the later frames. Therefore, the detected observation is already associated with that particular track. During the initial stage of tracking, a new track can be initialized by the user or by using non-recognition-based detection. The features of the tracked object are then learned for recognizing it in later frames.

Since illumination change is hard to model with a single colour model, we implemented two colour models for PBOD—RGB and HSV—where the latter is heavily used during illumination change. For histogram matching, we explored five histogram similarity modelling: correlation [7], chi squared [8], intersection [9], Bhattacharyya [10] and Poisson distribution [11]. We reviewed and compared two schemes for PBOD [11], one deterministic and the other probabilistic. The former method relies on threshold-based decision making while the latter method implements maximum a posterior and Bayes risk for decision-making. Both algorithms share the same basic structure but differ in whether patch matching and smoothing procedures are deterministic or probabilistic. Details of the algorithms are explained in the methodology section. The main novelties of PBOD are (1) the fusion between a feature and template-based approach; (2) modelling histogram similarity by Poisson distribution; (3) probabilistically adjusted patch position and size to fit the tracked object completely. Finally, the output patch is fed into any filter-based tracker as the measurement input.

This paper is organized into 6 sections. A literature review will be presented in Section 2. Details of the algorithms are fully explained in Section 3, where each subsection explains in detail the methods to obtain points of interest, generate candidate patches, find the best patch, perform position alignment and adjust the patch's size. In Section 4, the pseudo-codes of both deterministic and probabilistic PBOD are given for more clarity. Simulation results and discussions are presented in Section 5. The conclusions are given in the last section to emphasize the performance difference.

## 2. Literature Review

Since we are focusing on obtaining an observation through recognition, the two most common methods of object recognition are the template- and feature-based methods. The feature-based approach usually recognizes the object by obtaining a match based on the feature descriptor. The template-based approach uses the shape or a collection of pixels in finding a match. The major trade-offs of both approaches are the distinctiveness property and the generalization property. Most feature-based approaches have high distinctiveness property but low in generalization property. This explains why feature-based approaches do not work well for blurred images but perform exceptionally well on rich textured objects. On the other hand, the template-based approach has low distinctiveness property but is very good in generalizes the object detection. Even blurred and non-rigid objects can still be recognized reliably.

Template-based recognition is an approach that requires a database or a collection of possible templates to be built before any matching can be performed. This method is used in many license plate recognition systems [12–14]. Templates of the characters are built under several viewing conditions before matching is performed. Another popular template-based system is human recognition such as the work by Hsieh *et al.* [15]. The authors divided the frames into nine sections where each section has a different database of the human silhouette. It reduced processing time significantly by anticipating the human shape at the selected viewing angle. This clever approach allows the system templates to capture all possible transformations in a smaller database resulting in acceptable processing time. Sometimes, the templates or databases are obtained by extensive training even to the extent of 80 million images [16]. Simple template-based recognition is demonstrated by Bradski and Kaehler in [7], where they slide a single template across the whole image. The main advantage of the template-based approach is that it manages to recognize the object under moderate deformation, blurring and illumination change, all of which are common in video applications. An example template matching technique built for video application is the system by Cole *et al.* [17], where they used an adaptive scaling technique to reduce computation burden. The authors also reduce the number of matching candidates by segmenting the database into several classes and further breaking it down within a class into several groups. Yan *et al.* [18] then proposed a sub-template mean-shift method. *m*-best templates were chosen as the candidate based on their distance from the tracked object. This spatial distance also signified the a priori knowledge, which a Gaussian-based voting is employed to select the best template.

One of the most cited paper regarding point-based detector is Scale Invariant Feature Transform (SIFT) [19], which generates robust features that works exceptionally well even for problems of rotation, scaling and moderate illumination change. Ke and Suthankar [20] improved SIFT's feature distinctiveness property by applying principal components analysis. Later, Burghouts and Geusebroek [21] fused SIFT with a colour invariance [22] algorithm to achieve robustness to illumination changes. Their algorithm transformed the image into colour invariant forms, which they divided into **E**, **W**, **C** and **H** colour invariants before applying SIFT. Performance evaluations of SIFT and its variants are explained in depth by Mikolajczyk and Schmid [23]. They also introduced their own algorithm, GLoH, which improved SIFT by incorporating more spatial properties during histogram accumulation. In 2004, Ledwich and Williams [24] proposed a simplified SIFT feature for reducing computational burden. The author claimed that a significant gain in processing speed is obtained at minimal accuracy loss by utilizing structural similarity to reduce the number of keypoints generated. Moreover, Bay *et al.* [25] introduced Speeded Up Robust Features (SURF), which claims similar detection performance to SIFT but requires less computational burden. The underlying principals used in SURF are the combination of integral image and Hessian matrix based point descriptors.

Another method of obtaining tracking observation is by applying a histogram-based method. Histogram-based tracking algorithms [3,26] have been applied successfully to non-rigid objects because the matching is done based on the statistics of a group of pixels. A multilevel thresholding of the histogram data is used by Chen *et al.* [27] to improve the detection robustness to illumination changes and spurious infrared noise. The most popular histogram-based tracker is a mean shift algorithm [28] where the next location is predicted based on the input of histogram backprojection via the mean shift algorithm. Bradski introduced CAMSHIFT [26], which integrates scalability into the mean shift algorithm, thus allowing the tracked object to have variable size. A kernel-based tracker that utilizes the Epanechnikov kernel profile has been introduced by Comaniciu *et al.* [3]. This approach puts more emphasize on pixels that are closer to the anchor pixel and less weight on distant pixels for accumulating the histogram's bin values. They also apply the Bhattacharyya distance [10] for measuring similarity between two histograms.

In general, a kernel-based algorithm performs well for single object tracking [3,29,30]. However, as the scene becomes crowded and more objects need to be tracked, the algorithms start to falter, especially during occlusion, poor segmentation and inability to segment touching detections. The works by Namboodiri *et al.* [31] and Peng *et al.* [32] attempted to solve the problem of occlusion. The algorithm of Namboodiri *et al.* tweaks the localization of the mean shift by applying both forward and reverse methods so that it converges to the true modes. They also add scalability by utilizing SIFT's scale. However, this contradicts their assertion that their algorithm should require less processing power as it is a known fact that SIFT uses more processing power compared with the mean shift methods. The work by Peng *et al.* focuses on how to improve the updating method for the object model, for which they utilize a Kalman filter prediction method. The predicted object model is called the candidate model while the previous model is called the current model. Hypothesis testing is used to select the correct histogram model. In their paper, Leichter *et al.* [33] improved the kernel-based method by using multiple models for the object so that it tracks well under sudden changes of viewpoint. This method requires the user to initialize the object model in several views, which can be quite problematic. The main weakness of the mean shift algorithm is it depends on the proximity property where it is prone to failure when the object's movement is fast. The algorithm of Li *et al.* [30] approaches this problem by extending the search area based on their hexagon method. However, it is a brute force search that requires a significant amount of processing. Besides, the algorithm fails if the object moves fast enough such that it leaves their search region.

## 3. Patch-Based Observation Detection (PBOD): Deterministic and Probabilistic Approaches

**Figure 1.**
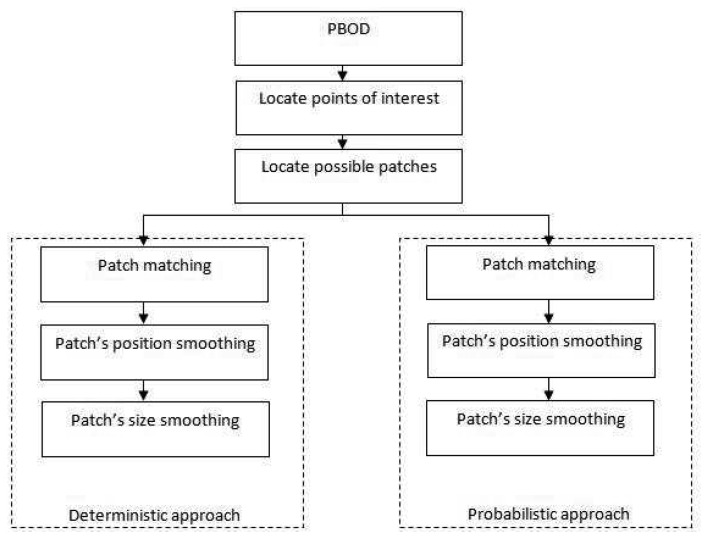
Block diagram of deterministic and probabilistic PBODs.

Both methods were first introduced in [11]. PBOD is built specifically for obtaining tracking observations in challenging surroundings and environment. This algorithm has a moderate distinctiveness property with better recognition accuracy for most applications. The distinctiveness property is the measure of the uniqueness of a descriptor, while generalization property is the ability to find a match in a noisy environment. A typical example is if the object is blurred, the descriptor will not be able to match the points of interest. However, if the generalization property is good, the algorithm can still find a match by generalizing the information of the object. The main challenge lies in recognizing the objects under illumination change, blurring effects, moderate deformation and non-rigid objects, low ambient illumination and objects with homogenous texture. We have developed two versions of the observation detector, the deterministic PBOD and the probabilistic PBOD. Both approaches share the same algorithm up to the possible patch generation. For the latter stage, the algorithms differ as indicated by the name where decisions are made deterministically or probabilistically. The main five components of the algorithm are shown in Figure 1.

### 3.1. Generate Points of Interest and Possible Patches

A point of interest is used to obtain the location to generate vector descriptors. These descriptors are matched between frames for building possible patches. The original location of the object or the original patch in the first frame is initialized by the user. The importance of this user defined patch is that it serves as the reference for building the statistical data used in matching and smoothing procedures and in particular, the reference histograms. Moreover, the size of the previous frame patch indicates the object's size. Let *β_w_* and *β_h_* denote the width and height of a bounding box or patch. For this section, both current $F_{R,G,B}^{t,x,y}$ and previous frames $F_{R,G,B}^{t-1,x,y}$ are transformed to grey scale space $\left(F_{I}^{t,x,y},F_{I}^{t-1,x,y}\right)$. Corner detectors as defined by Shi and Tomasi [34] are applied to find the possible points of interest. The threshold used in the Shi and Tomasi algorithm is around 0.01, which signifies the minimum eigenvalue threshold required for the point to be considered as a corner. This corner detector was selected because of its ability to generate points even under low ambient illumination and for low textured objects. For the previous frame, the points are generated inside the predefined patch only, while for the current frame, the points are generated for the entire image.

**Figure 2.**
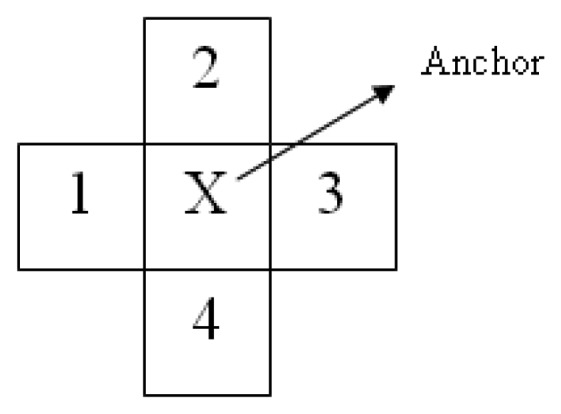
Neighbourhood pattern used for vector generation.

Then, vector descriptor **V***^t,x,y^* is used to match the points of interest between frames. Possible bounding boxes are generated at each corner where the vectors are matched. We use the RGB colour model, and each channel is treated separately. Therefore, there will be three sets of vectors for each point of interest, ${\text{V}}_{R}^{t,x,y}$, ${\text{V}}_{G}^{t,x,y}$, ${\text{V}}_{B}^{t,x,y}$. The vectors are generated by finding the colour difference between the anchor pixel and its selected neighbouring pixels as shown in Figure 2. Let *i* denote the colour channel and *i* ∈ {*R*, *G*, *B*}.

(1) $${\text{V}}_{i}^{t,x,y}=\{F_{i}^{t,x-1,y}-F_{i}^{t,x,y},F_{i}^{t,x,y-1}-F_{i}^{t,x,y},F_{i}^{t,x+1,y}-F_{i}^{t,x,y},F_{i}^{t,x,y+1}-F_{i}^{t,x,y}\}$$

Each of the vector components is sorted from the lowest to the highest value. The reason for sorting is to account for the rotation of the object, while the use of colour differences allows the algorithm to find good points of interest even during an illumination change. This algorithm does not have rotation invariant abilities since all matchings are done based on the colour histogram. The reason for using 4-connected neighbourhood data instead of 8-connected neighbourhood is to produce as much as possible candidate patches in the early stage, which later will be filtered by the subsequent processes. Each vector from the initial frame is compared with each vector in the next frame. The decision rule $L_{1}^{t,x,y}$ for matching the vectors is shown in Equation (2) where the differences between each vector component are summed up, and the final value is obtained by combining all three channels' differences, *i.e.*, a Manhattan distance. Then it is compared with a predefined threshold, 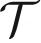
_1_, which was found from experiments to be optimal in the range 11 to 13. Let $L_{2}^{t,x,y}$ denote the label which is set to one when the vectors are matched and zero for unmatched vectors.

(2) $$L_{1}^{t,x,y}=\sum_{\forall \;i}\left|{\text{V}}_{i}^{t,x,y}-{\text{V}}_{i}^{t-1,x,y}\right|,i\in \{R,G,B\}$$

(3) $$L_{2}^{t,x,y}=\{\begin{array}{c} 1\;\text{if}\;L_{1}<{𝒯}_{1}\\ 0\;\text{if}\;L_{1}\geq {𝒯}_{1} \end{array}$$

**Figure 3.**
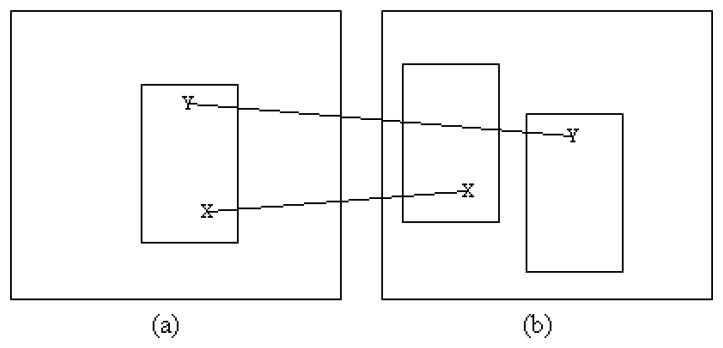
Examples of constructing new patches between the frames. The bounding boxes are aligned with respect to the matched vectors in the first frame. (**a**) First frame (**b**) Second frame.

All the matched vectors are candidates for locations at which patches are built. Patches for the second frame are generated around the location of the matched vector in the first frame with respect to the original bounding box. Figure 3 shows an example of how the bounding box is generated. Initially, the size of the object is assumed to remain constant between frames.

A subsequent test for distinguishing overlapping patches is performed after all patches have been assigned location and size. This is performed due to different matched features possibly lying close to each other. If the difference is small, the patch should be smoothed out as one. This is done in order to reduce the calculation burden by reducing the number of patches. Moreover, most of the small differences occur because of “noise” in the patch generation process. The decision rule *L*_3_ for determining overlapping patches is calculated as in Equation (4). Patch smoothing to combine all patches that lie close to each other is performed if the overlapping area *O_a_* is more than 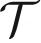
_2_% of the original patch size.

(4) $$L_{3}=\{\begin{array}{c} 1\;\text{if}\;O_{a}>({𝒯}_{2}\times \beta_{w}\times \beta_{h})\\ 0\;\text{if}\;O_{a}\leq ({𝒯}_{2}\times \beta_{w}\times \beta_{h}) \end{array}$$

**Figure 4.**
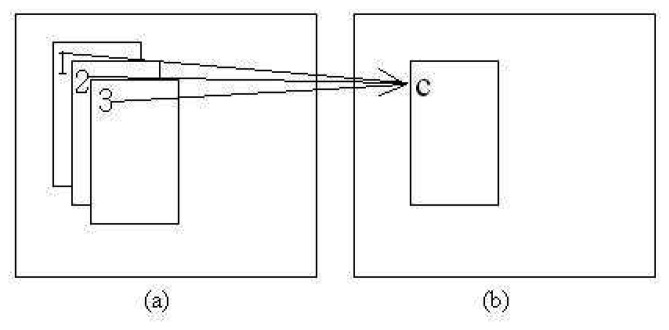
Example of several patches combination. (**a**) Original patches (**b**) Combined patch.

The new combined patch location, (*x̄*, *ȳ*) is the average of the corresponding centers of the overlapping patches as shown in Figure 4 where *N_L_* is the number of *L*_3_s detected. Comprehensive pseudo-code of both points of interest and possible patches generation are given in Algorithm 1.

(5) $$\beta^{\bar{x},\bar{y}}=\left(\frac{1}{N_{L}}\sum_{i=1}^{N_{L}}\beta^{x_{i}},\frac{1}{N_{L}}\sum_{i=1}^{N_{L}}\beta^{y_{i}}\right)$$

**Algorithm 1 Generate points of interest and possible patches**
**Require:**Points of interest(a) Obtain user input bounding box during the first frame(b) Convert previous and current frames to grey scale for each cycle(c) Perform Smoothing to both frames with Gaussian convolution(d) Find Laplacian for each frame(e) Obtain the matrix 
$\left[\begin{array}{cc} F_{I_{xx}}^{i,x,y} & F_{I_{xy}}^{i,x,y}\\ F_{I_{yx}}^{t,x,y} & F_{I_{yy}}^{t,x,y} \end{array}\right]$ for each pixel(f) **If** min eigenvalue> 

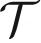
  accept the point as possible POI **Else**  reject the point **End If****Require:**tentative patches(a) Each channel of RGB is treated separately(b) Built vector descriptors, 
${\text{V}}_{i}^{t,x,y}$ for both frames(c) **For**
*i*, …, total POI **Do**
$L_{1}=\sum_{\forall \;i}\left|{\text{V}}_{i}^{t,x,y}-{\text{V}}_{i}^{t-1,x,y}\right|$ **End For**(d) Obtain *L*_2_ to decide either to accept the POI or not **If**
*L*_1_ < 

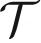
_1_
$L_{2}^{t,x,y}=1$ **Else**
$L_{2}^{t,x,y}=0$ **End If**(e) Construct a patch at each point where *L*_2_ = 1 The original size of each patch is similar with the final size of the previous frame(d) Smooth out overlapping patches by combining redundant patches **IF**
*O_a_* > (

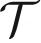
_2_ × *β_w_* × *β_h_*)  combine the patches by finding the mean of the patches **Else**  remain as it is **End If**


### 3.2. Patch Matching

Patch matching is performed to find the patch where the object most likely resides. The match is done by comparing the histograms of previous and current frame patches. Two colour models are considered, *i.e.*, RGB and HSV. For the case of constant illumination, the RGB colour model gives better histogram comparison than does HSV. The RGB colour model is better due to its more distinctive feature when there is no illumination change. If illumination change occurs, the hue channel from the HSV colour model gives better comparison performance. This is because the hue channel is more stable under moderate illumination change even though its distinctive property degrades. Another reason for choosing HSV colour model is due its simplicity, which results in low computational burden as compared with other colour invariant models such as [22,35]. For RGB colour space, a 3-dimensional histogram is built for each patch while a 1-dimensional histogram is built for the hue channel. The conversions from RGB model to HSV model are given by Tsai [36].

(6) $$V=\frac{1}{3}(R+G+B)$$

(7) $$S=1-\frac{3}{R+G+B}\min(R,G,B)$$

(8) $$H=\{\begin{array}{l} \theta \;\text{if}\;B\leq G\\ 360{}^{\circ}-\theta \;\text{if}\;B>G \end{array}$$

where

(10) $$\theta =\cos^{-1}\left(\frac{\frac{1}{2}[(R-G)+(R-B)]}{\sqrt{{\left(R-G\right)}^{2}+(R-B)(G-B)}}\right)$$

#### 3.2.1. Deterministic Approach

After the number of patches has been finalized, histogram correlation ( 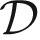
_
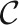
_) between current frame patches and previous frame patch is used to identify the object. The test is divided into two levels, where the first level is used to obtain the match under normal illumination, while the second-level test is initiated when an illumination change is detected. The first-level test depends on RGB colour space while a 1-dimensional hue histogram is used for the second level. Let *N_b_* be the number of histogram bins in one dimension while previous and current frames' histograms are denoted by *n* and *m* respectively. *m_i_* is the value of *i^th^* bin of the current frame histogram where each histogram is normalized first before matching them. The output range is [−1, 1], where 1 indicates a perfect match while −1 signifies a total mismatch. If ( 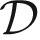
_
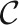
_) is zero, it signifies a very low correlation value that indicates an illumination change has occurred or no match is found.

For a 1-dimensional histogram:

(11) $${𝒟}_{c_{1}}(n,m)=\frac{\sum_{i=1}^{N_{b}}n_{i}m_{i}-\frac{\sum_{i=1}^{N_{b}}n_{i}\sum_{i=1}^{N_{b}}m_{i}}{N_{b}}}{\sqrt{\left(\sum_{i=1}^{N_{b}}{\left(n_{i}\right)}^{2}-\text{K}).(\sum_{i=1}^{N_{b}}{\left(m_{i}\right)}^{2}-\text{L}\right)}}$$

where

(12) $$\text{K}=\frac{{\left(\sum_{i=1}^{N_{b}}n_{i}\right)}^{2}}{N_{b}},\text{and}\;\text{L}=\frac{{\left(\sum_{i=1}^{N_{b}}m_{i}\right)}^{2}}{N_{b}}$$

and for a 3-dimensional histogram:

(13) $${𝒟}_{c_{3}}(n,m)=\frac{\sum_{i=1}^{N_{b}}\sum_{j=1}^{N_{b}}\sum_{k=1}^{N_{b}}n_{i,j,k}m_{i,j,k}-\frac{\sum_{i=1}^{N_{b}}\sum_{j=1}^{N_{b}}\sum_{k=1}^{N_{b}}n_{i,j,k}\sum_{i=1}^{N_{b}}\sum_{j=1}^{N_{b}}\sum_{k=1}^{N_{b}}m_{i,j,k}}{3N_{b}}}{\sqrt{\left(\sum_{i=1}^{N_{b}}\sum_{j=1}^{N_{b}}\sum_{k=1}^{N_{b}}{\left(n_{i,j,k}\right)}^{2}-\text{K}).(\sum_{i=1}^{N_{b}}\sum_{j=1}^{N_{b}}\sum_{k=1}^{N_{b}}{\left(m_{i,j,k}\right)}^{2}-\text{L}\right)}}$$

where

(14) $$\text{K}=\frac{{\left(\sum_{i=1}^{N_{b}}\sum_{j=1}^{N_{b}}\sum_{k=1}^{N_{b}}n_{i,j,k}\right)}^{2}}{3N_{b}},\text{and}\;\text{L}=\frac{{\left(\sum_{i=1}^{N_{b}}\sum_{j=1}^{N_{b}}\sum_{k=1}^{N_{b}}m_{i,j,k}\right)}^{2}}{3N_{b}}$$

Since some of the matched vectors are found near the border of the image, certain patches may have some regions with components outside the frame. In this situation, we set out of bound components to be low (black), which consequently increases the probability of detecting occlusion. The patch with the highest correlation, $\beta_{1}^{\max}$, is taken as the candidate for the object location. However, $\beta_{1,R,G,B}^{\max}$ should exceed a predefined threshold 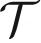
_3_ or otherwise the second-level test is initiated. The optimal value for 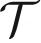
_3_ is found from extensive simulations to be around 0.73. Let *ε_d_* be the indicator, which takes the value 1 if the first-level test is satisfied and 0 if the second-level test is initiated.

(15) $$\varepsilon_{d}\;=\{\begin{array}{l} 1(1^{\text{st}}\;\text{level test})\;\text{if}\;\beta_{1,R,G,B}^{\max}>{𝒯}_{3}\\ 0(2^{\text{nd}}\;\text{level test})\;\text{if}\;\beta_{1,R,G,B}^{\max}\leq {𝒯}_{3} \end{array}$$

**Figure 5.**
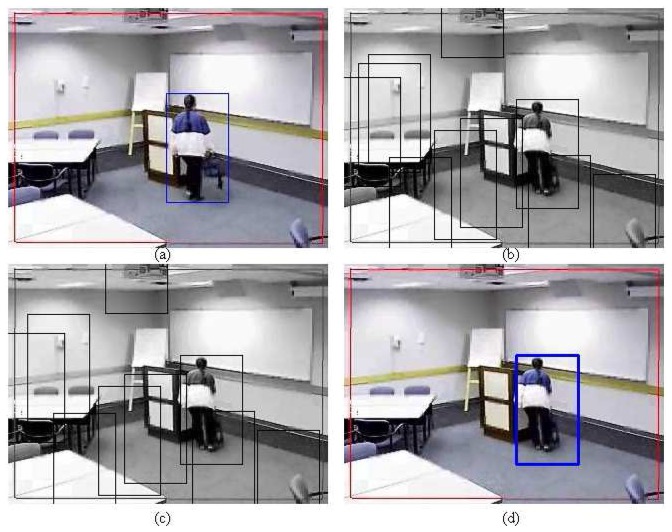
Procedures for selecting the right patch. (**a**) Original patch (**b**) Raw patches (**c)** Combined patches (**d**) Maximum correlation patch.

For the second-level test, both previous and current frames are transformed from RGB to HSV colour model. Only the hue channel is utilized where the illumination has changed due to the previous assumption. The reason for utilizing only hue information under illumination change is because of its stability compared with other colour information [7]. By using the same set of possible patches as in the first-level test, the hue histogram of each patch is obtained. Then, each correlation value is calculated using Equation (11) and the patch with the maximum correlation is taken as the candidate for the object location. The maximum correlation is compared with the threshold value 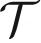
_4_ in order to determine if the object still resides in the frame or not. Let *L*_4_ represent the label of detecting the object. Figure 5 shows an example of selecting the right patch between two consecutive frames.

(16) $$L_{4}=\{\begin{array}{l} 1\;(\text{object is detected})\;\text{if}\;\beta_{2,H}^{\max}>{𝒯}_{4}\\ 0\;(\text{object have leaved the frame})\;\text{if}\;\beta_{2,H}^{\max}\leq {𝒯}_{4} \end{array}$$

(17) $$\beta_{3}=\{\begin{array}{l} \beta_{2,H}^{\max}\;\text{if}\;\varepsilon_{d}\;=\;0\\ \beta_{1,R,G,B}^{\max}\;\text{if}\;\varepsilon_{d}\;=\;1 \end{array}$$

#### 3.2.2. Probabilistic Approach

For the probabilistic approach, histogram matching is done by modelling the relationship between two histograms as a Poisson distribution as in Equations (18) and (19).

For a 1-dimensional histogram:

(18) $${𝒟}_{P_{1}}(n,m)=\prod_{i=1}^{N_{b}}\left(\frac{\exp^{-n_{i}}n_{i}^{m_{i}}}{m_{i}!}\right)$$

and for a 3-dimensional histogram:

(19) $${𝒟}_{P_{3}}(n,m)=\prod_{i=1}^{N_{b}}\prod_{j=1}^{N_{b}}\prod_{k=1}^{N_{b}}\left(\frac{\exp^{-n_{i,j,k}}n_{i,j,k}^{m_{i,j,k}}}{m_{i,j.k}!}\right)$$

A maximum likelihood approach is used to find the matched patch for both colour models. The likelihoods are modelled by Equations (18) and (19) where *β*_5_ denotes the matched patch and **x** represents the observation.

(20) $$P_{1}(\text{x}|\beta_{4})=\{\begin{array}{c} {𝒟}_{P_{1}}(n,m)\;\text{for HSV colour model}\\ {𝒟}_{P3}(n,m)\;\text{for RGB colour model} \end{array}$$

(21) $$\beta_{5}=\underset{\forall \;\beta_{4}}{\text{argmax}}\;P_{1}(\text{x}|\beta_{4})$$

There are two candidates for the most likely patch. The decision to choose the hue colour model over RGB is made by using a Neyman–Pearson hypothesis test [37]. Let *P***(x**; *H*_0_) = *P*_2_(*β*_4_,*_R_*,*_G_*,*_B_*), *P*(**x**; *H*_1_) = *P*_3_(*β*_4_,*H*) and *η*_1_ represent the threshold for the Neyman–Pearson hypothesis test. If the test favours *H*_0_, *ε_p_* is initialized as one. On the other hand, if *H*_1_ is chosen, *ε_p_* is set equal to zero. The parameter *ε_p_* indicates which colour space is used for position and size smoothing. The resultant patch *β*_6_ from the test will be the final matched patch.

(22) $${\text{NP}}_{1}=\frac{P_{3}(\beta_{4,H})}{P_{2}(\beta_{4,R,G,B})}>\eta_{1}$$

(23) $$\beta_{6}=\{\begin{array}{l} \beta_{4,R,G,B}\;\text{if}\;P_{3}(\beta_{4,H})<\eta_{1}P_{2}(\beta_{4,R,G,B})\\ \beta_{4,H}\;\text{if}\;P_{3}(\beta_{4,H})\geq \eta_{1}P_{2}(\beta_{4,R,G,B}) \end{array}$$

### 3.3. Position Smoothing

Position smoothing is used to adjust the patch's centroid to precisely fit the object's centroid. Sometimes, the calculated patch is slightly misaligned with the tracked object. This error is prevalent during illumination change and low ambient illumination. The adjustment is divided into two cases depending on the value of *ε*. The step size *δ* used for adjusting the patch translation is determined first. Let 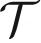
_5_ denote a weight factor that takes values in [0 1].

(24) $$\delta ={𝒯}_{5}(\min(\beta_{w},\beta_{h}))$$

#### 3.3.1. Deterministic Approach

**Figure 6.**
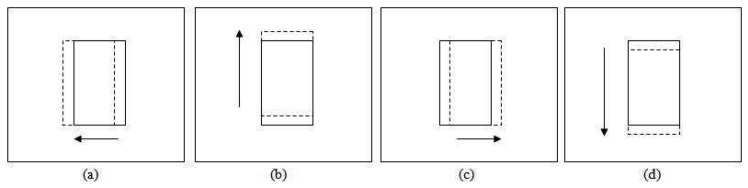
Patches coordination for location smoothing (**a**) Left side translation (**b**) Upward translation (**c**) Right side translation (**d**) Downward translation.

The translation test for adjusting the patch location is performed in four directions as shown in Figure 6, *i.e.*, leftward $\left(\beta_{7}^{a}\right)$, upward $\left(\beta_{7}^{b}\right)$, rightward $\left(\beta_{7}^{c}\right)$ and downward $\left(\beta_{7}^{d}\right)$. The solid line patch is the original position while the dashed line patch is the patch translated by a step size value. The statistical properties of these patches are retrieved depending on the *ε_d_* value. For *ε_d_* equal to 1, the RGB histograms for each patch are built and its correlation with the previous frame patch's histograms is calculated.

Every correlation ( 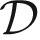
_
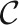
_) of the four new patches $\left(\beta_{7}^{a},\beta_{7}^{b},\beta_{7}^{c},\beta_{7}^{d}\right)$ and the original patch (*β*_6_) correlation ( 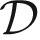
_
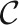
_^current^) are compared. The new patch location is selected based on the maximum correlation 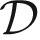
_
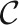
_^max^ among them. If the original patch correlation is the maximum, the position will remain the same. If any of the new patch's correlation is the maximum, the detected patch is shifted toward that corresponding direction. The new maximum correlation is reset when the patch is moved. The procedures are repeated until the maximum correlation among the new patches is less than the original patch correlation. 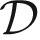
_
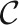
_^max^ is stored for later usage during the shrinkage and expansion test.

(25) $${{𝒟}_{c}}^{\max}=\max\{{{𝒟}_{c}}^{a},{{𝒟}_{c}}^{b},{{𝒟}_{c}}^{c},{{𝒟}_{c}}^{d}\}$$

(26) $$\beta_{8}=\{\begin{array}{c} \beta_{6}\;\text{if}\;{{𝒟}_{c}}^{\max}>{{𝒟}_{c}}^{\text{ori}}\\ \beta_{7}^{\max}\;\text{if}\;{{𝒟}_{c}}^{\max}\leq {{𝒟}_{c}}^{\text{ori}} \end{array}$$

For the case of *ε_d_* equal to 0, only the hue channel histogram is generated instead of RGB histograms. The remaining steps follow the same procedure as before; the correlation is calculated and used for the position smoothing comparison. All four new patch locations are tested, and the stopping criterion is when the maximum hue correlation among the new patches is less than the original patch correlation.

#### 3.3.2. Probabilistic Approach

In the probabilistic approach, the same four new candidate patches are created for adjusting the patch position as shown in Figure 6, representing translations in four directions of the pivot patch (*β*_6_)— leftward $\left(\beta_{9}^{a}\right)$, upward $\left(\beta_{9}^{b}\right)$, rightward $\left(\beta_{9}^{c}\right)$ and downward $\left(\beta_{9}^{d}\right)$. Histograms of each of the five patches, including the original position patch are obtained, and the maximum likelihood is used to find the new location. Likelihood is derived from the relationship between the previous and current frames' histogram as in the Equations (18) and (19). Let *β*_10_ denote the output of position smoothing.

(27) $$P_{4}(\text{x}|\beta_{9})=\{\begin{array}{c} {𝒟}_{P_{1}}(n,m)\;\text{if}\;\in_{p}=0\\ {𝒟}_{P_{3}}(n,m)\;\text{if}\;\in_{p}=1 \end{array}$$

(28) $$\beta_{10}=\underset{\forall \beta_{9}^{i}}{\text{argmax}}\;P(\text{x}|\beta_{9}),i\in \{a,b,c,d\}$$

For each iteration, the pivot position is reinitialized by letting *β*_6_ = *β*_10_, so that all four new translated patches for the next iteration are built around *β*_10_. The algorithm is iterated until the estimated patch position remains the same as shown by the decision rule *L*_5_.

(29) $$L_{5}=\{\begin{array}{l} 0\;(\text{stop the iteration})\;\text{if}\;\beta_{10}=\beta_{6}\\ 1\;(\text{continue the iteration})\;\text{if}\;\beta_{10}\neq \beta_{6} \end{array}$$

### 3.4. Size Smoothing

**Figure 7.**
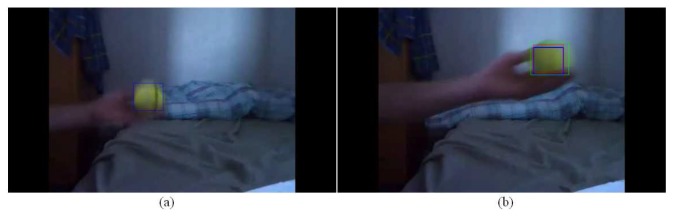
Sample output of position and size smoothing algorithm (**a**) Original object (**b**) Blue box: Output of patch matching, Red box: Output of position smoothing and Green box: Output of size smoothing.

**Figure 8.**
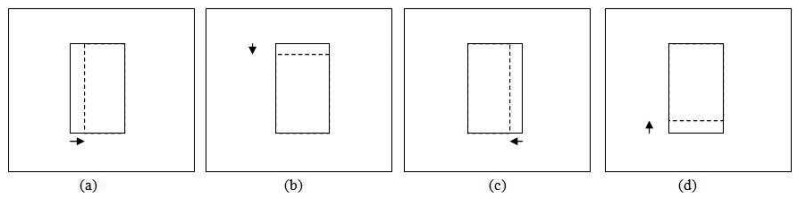
Patterns for shrinkage patch (**a**) Left side shrinkage (**b**) Upper side shrinkage (**c**) Right side shrinkage (**d**) Lower side shrinkage.

**Figure 9.**
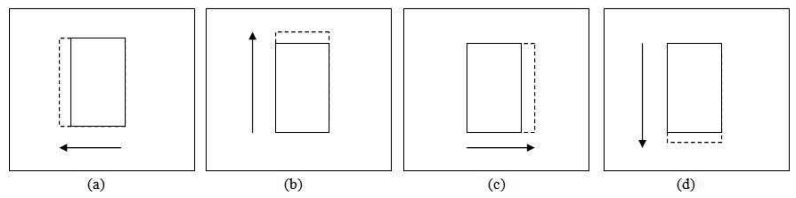
Patterns for expansion patch (**a**) Left side expansion (**b**) Upper side expansion (**c**) Right side expansion (**d**) Lower side expansion.

This section focuses on adjusting the size of the patch so that it provides a good fit to the tracked object. Generally, the apparent size of the object becomes bigger as it moves closer to the camera and smaller as it moves away. However, size increment and decrement between consecutive frames should not be very large. Based on this assumption, we limit the scale change for size smoothing by at most a factor of $\frac{1}{2}$ between two consecutive frames. Figure 7 shows an example of applying size smoothing in PBOD. The algorithm is still divided into two sections, which depend on the *ε* value as the colour space and parameters used are different. Eight new patches with different sizes are utilized for the size smoothing test. The same *δ* used in position smoothing is applied to adjust the patch size. Four shrinkage and four expansion pattern patches are obtained by either subtracting from or adding to one of the patch corners by a step size value. Figure 8 shows the shrunk patches $\left(\beta_{11}^{a},\beta_{11}^{b},\beta_{11}^{c},\beta_{11}^{d}\right)$, while the expanded patches are shown in Figure 9 $\left(\beta_{12}^{a},\beta_{12}^{b},\beta_{12}^{c},\beta_{12}^{d}\right)$.

#### 3.4.1. Deterministic Approach

We first consider the case where *ε_d_* equals to one in which RGB channels are used for building the histogram. A parameter *α* is calculated as the weight in determining the size pattern.

(30) $$\alpha =0.1\times (1-{{𝒟}_{c}}^{\max})$$

A test to determine the size pattern is performed to find out whether the object is expanding or shrinking. Here, only shrinkage patterns are considered. RGB histograms are generated for all new shrinkage patterns. Then, the histogram's size is normalized before correlations between the new patches and the anchor patch *β*_8_ are calculated. Let *N_β_*_1_ and *N_β_*_2_ denote the number of pixels inside patches from the previous and current frames respectively. Each histogram bin value *H* is adjusted by the ratio of *N_β_*_2_ to *N_β_*_1_.

(31) $$H^{\text{new}}=\left(\frac{N_{\beta 2}}{N_{\beta 1}}\right)H^{\text{old}}$$

The average correlation among the channels for each patch is calculated. The weighted correlation $\hat{{𝒟}_{c}}$ between the new shrinkage patches are used to determine the size pattern. The weight 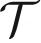
_6_ is used to find $\hat{{𝒟}_{c}}$, which are comprised of *D_C_*^max^ and the average of *D_C_* of the shrinkage patches.

(32) $$\hat{{𝒟}_{c}}={𝒯}_{6}({{𝒟}_{c}}^{\max})+(1-{𝒯}_{6})({\bar{𝒟}}_{c})$$

Then the weighted correlation is compared with the maximum correlation (*D_Cβ_*_8_) from the location smoothing to determine the size pattern, *L*_6_.

(33) $$L_{6}=\{\begin{array}{c} -1\;\text{if}\;\hat{{𝒟}_{c}}>{𝒟}_{c\beta_{8}}\\ 1\;\text{if}\;\hat{{𝒟}_{c}}\leq {𝒟}_{c\beta_{8}} \end{array}$$

(34) $$L_{7}=\{\begin{array}{c} 0\;\text{if}\;{{𝒟}_{c}}^{\beta_{i}}<({{𝒟}_{c}}^{\max}-\alpha )\\ 1\;\text{if}\;{{𝒟}_{c}}^{\beta_{i}}\geq ({{𝒟}_{c}}^{\max}-\alpha ) \end{array},\beta_{i}\in \{\beta_{12}^{a},\beta_{12}^{b},\beta_{12}^{c},\beta_{12}^{d}\}$$

**Algorithm 2 Deterministic PBOD**
**Require:**patch matching(a) For each patch, obtain 

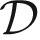
_
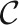
3_ based on RGB space(b) **If**
$\beta_{1,R,G,B}^{\max}>{𝒯}_{3}$  *ε_d_* = 1 **Else**  *ε_d_* = 0 **End If**(c) **If**
*ε_d_* = 0, start 2*^nd^* level test(d) For 2*^nd^* level test, obtain 

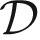
_
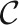
1_ for each patch(e) Select 
$\beta_{2,H}^{\max}$ as the candidate patch**Ensure:**position smoothing(a) Select colour space based on *ε_d_*, either RGB or HSV(b) Determine the step size, *δ*(c) Construct the translated patch, 
$\beta_{7}^{a},\beta_{7}^{b},\beta_{7}^{c},\beta_{7}^{d}$(d) Find maximum 

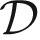
_
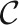
_(e) **While**
*β*_8_ = *β*_6_
**Do** **If**

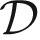
_
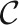
_^max^ > 

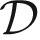
_
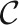
_^ori^  *β*_8_ = *β*_6_ **Else**  
$\beta_{8}=\beta_{7}^{\max}$ **End If****End While****Ensure:**size smoothing(a) Determine *α* as the size factor(b) Obtain size pattern based on *β*_9_ histograms either to use shrinkage or expansion pattern(c) Normalize histograms size for fairer histogram matching(d) **If**
$\hat{{𝒟}_{c}}>{𝒟}_{c\beta_{8}}$  Utilize shrinkage patch patterns **Else**  Utilize expansion patch patterns **End If**(e) **Switch**  **Case** (shrink)   **If**

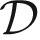
_
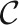
_^*i*^ < (

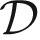
_
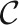
_^max^ − *α*), *L*_13_ = 0 **Else**
*L*_13_ = 1  **Case** (expand)   **If**

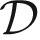
_
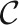
_*^j^* < (

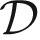
_
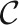
_^max^ + *α*), *L*_14_ = 0 **Else**
*L*_14_ = 1 **End Switch**(f) Reiterate the process until the patch has converged to a certain size or number of iteration has exceeded *p* cycles.


For the shrinkage pattern, the patches used are shown in Figure 9. The same steps used in the expansion pattern are applied but with a different decision rule *L*_8_ as shown in Equation (35). A more stringent threshold is used in the shrinkage test is to counter the homogenous texture problem. This is because the test will give good correlation even though the object size is not shrinking.

(35) $$L_{8}=\{\begin{array}{c} 0\;\text{if}\;{{𝒟}_{c}}^{\beta_{j}}<({{𝒟}_{c}}^{\max}+\alpha )\\ 1\;\text{if}\;{{𝒟}_{c}}^{\beta_{j}}\geq ({{𝒟}_{c}}^{\max}+\alpha ) \end{array},\beta_{j}\in \{\beta_{11}^{a},\beta_{11}^{b},\beta_{11}^{c},\beta_{11}^{d}\}$$

For the case of *ε_d_* equal to zero, the same set of algorithms are used but instead of RGB channels, only the hue channel is applied. The parameter *α* is replaced with *α*_1_ and *α*_2_. Both parameters are predefined values, as it is hard to find good closed form expression for them due to the complexity of the scene when the illumination changes. *α*_1_ is applied during the test for determining the size pattern, while *α*_2_ is applied during the shrinkage and expansion test. The algorithm stops when there is no size change or the iteration has exceeded two times. Full pseudo-code for the deterministic approach is given in Algorithm 2.

#### 3.4.2. Probabilistic Approach

For the probabilistic approach, *β*_10_ is used as the pivot point for creating all the new patches. A Bayesian approach is used to decide the final patch size *β*_13_ from among the nine patches, including the original patch *β*_10_.

(36) $$\begin{array}{cc} P_{5}(\beta_{11}|\text{x})=\frac{P_{5}(\text{x}|\beta_{i})P_{5}(\beta_{i})}{P_{5}(\text{x})}, & \beta_{i}\in \{\beta_{10},\beta_{11}^{a},\ldots ,\beta_{11}^{d},\beta_{12}^{a},\ldots ,\beta_{12}^{d}\} \end{array}$$

Since *P*(**x**) is equal for all nine patches:

(37) $$\begin{array}{cc} P_{5}(\beta_{11}|\text{x})\propto P_{5}(\text{x}|\beta_{i})P(\beta_{i}), & \beta_{i}\in \{\beta_{10},\beta_{11}^{a},\ldots ,\beta_{11}^{d},\beta_{12}^{a},\ldots ,\beta_{12}^{d}\} \end{array}$$

The value of *ε_p_* determines what type of histogram is built. If *ε_p_* equal to zero, a 1-dimensional hue histogram is used, while for *ε_p_* equal to one, a 3-dimensional RGB histogram is applied. Before any comparison is performed, the histogram size must first be normalized. The normalization of the histogram size follows the same Equation (31) as the deterministic case. Once again, the histogram relationship between the previous and the current frame patches are modelled by a Poisson distribution.

(38) $$P_{5}(\text{x}|\beta )=\{\begin{array}{c} P_{5}(n,m)\;\text{if}\;\varepsilon_{p}\;=\;0\\ P_{5}(n,m)\;\text{if}\;\varepsilon_{p}\;=\;1 \end{array}$$

Two sets of prior probabilities are used. These depend on whether the size of the detected object inclines towards expansion or shrinkage. The selection of a suitable prior probability is very important, as the likelihood of shrinkage is usually large even when the object expands. Thus, we apply lower prior probabilities to shrinkage candidates if the size is increasing. In order to determine which set of the prior probabilities to use, again a Neyman–Pearson hypothesis test is implemented, where *H*_0_ and *H*_1_ represent the expansion and shrinkage hypotheses. Only eight candidate patches are used (four shrinkage patches + four expansion patches) for this test where the same Poisson distribution as in Equation (38) is used. The maximum probability among the expansion patches represents the *H*_0_ probability, while the maximum probability among the shrinkage patches represents the *H*_1_ probability.

(39) $$\begin{array}{cc} {}[t]P(\text{x};H_{0})=\underset{\forall \;\beta^{i}}{\max}P_{5}(\text{x}|\beta_{i}), & \beta_{i}\in \{\beta_{12}^{a},\ldots ,\beta_{12}^{d}\} \end{array}$$

(40) $$\begin{array}{cc} P(\text{x};H_{1})=\underset{\forall \;\beta^{j}}{\max}P_{5}(\text{x}|\beta_{j}), & \beta_{j}\in \{\beta_{11}^{a},\ldots ,\beta_{11}^{d}\} \end{array}$$

Let *η*_2_ be the threshold for the Neyman–Pearson test.

(41) $${\text{NP}}_{2}=\frac{P(\text{x};H_{1})}{P(\text{x};H_{0})}>\eta_{2}$$

(42) $$P_{5}(\beta )=\{\begin{array}{c} P(\beta^{\text{expand}})\;\text{if}\;H_{0}\;\text{is true}\\ P(\beta^{\text{shrink}})\;\text{if}\;H_{1}\;\text{is true} \end{array}$$

After the prior probability is obtained, each of the nine patches posterior $\left(P_{5}(\beta_{10}|\text{x}),P_{5}(\beta_{11}^{a}|\text{x}),\ldots ,P_{5}(\beta_{11}^{d}|\text{x}),P_{5}(\beta_{12}^{a}|\text{x}),\ldots P_{5}(\beta_{12}^{d}|\text{x})\right)$ is calculated. Each side of the bounding box can be expanded or shrunk independently based on the *L*_9_ decision rule. Each side size is altered depending on whether the new posteriors exceed the original size posterior. *L*_9_ equal to one indicates that the size is updated, while *L*_9_ equal to zero indicates that the size remains constant.

(43) $$\begin{array}{cc} L_{9}=\{\begin{array}{c} 0\;\text{if}\;P_{5}(\beta_{10}|\text{x})\leq P_{5}(\beta_{i}|\text{x})\\ 1\;\text{if}\;P_{5}(\beta_{10}|\text{x})>P_{5}(\beta_{i}|\text{x}) \end{array}, & \beta_{i}\in \{\beta_{11}^{a},\ldots ,\beta_{11}^{d},\beta_{12}^{a},\ldots ,\beta_{12}^{d}\} \end{array}$$

**Figure 10.**
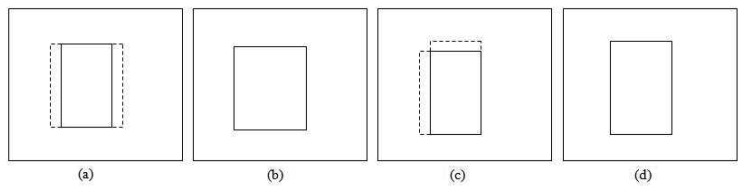
Example of the patch expansion (**a**)(**c**) Original patch (**b**) Result if the right and left side expansion are true (**d**) Result if the left and upper side expansion are true.

Probabilistic PBOD will follow the same rules as deterministic PBOD, which allows each side to be independently updated as shown in Figure 10. The iteration is terminated if no change is detected. Algorithm 3 denotes the pseudo-code of the probabilistic approach from patch matching to size smoothing.

## 4. Simulation Results and Discussion

The accuracy and effectiveness of PBOD were validated rigorously, in which the simulations are divided into three subsections:

1. Histogram matching performance
2. Deterministic and probabilistic PBOD
3. Probabilistic PBOD, Kernel tracker and SIFT-based tracker.

**Algorithm 3 Probabilistic PBOD**
**Require:**patch matching(a) Calculate 

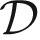
_
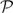
__1_ and 

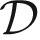
_
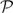
__3_ for all patches(b) Find *β*^max^ for both 

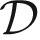
_
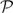
__1_ and 

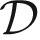
_
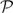
__3_(c) Apply Neyman–Pearson to decide between RGB and HSV colour space **If**
*H*_1_ is true  
$\beta_{6}=\underset{\forall \;\beta }{\text{argmax}}\;{𝒟}_{P_{3}}$ **Else**  
$\beta_{6}=\underset{\forall \;\beta }{\text{argmax}}\;{𝒟}_{P_{1}}$ **End If****Ensure:**position smoothing(a) Select colour space based on *ε_p_*(b) Determine the step size, *δ*(c) Construct the translated patch, 
$\left(\beta_{7}^{a},\beta_{7}^{b},\beta_{7}^{c},\beta_{7}^{d}\right)$(d) For each patch, histogram matching is modelled by Poisson distribution(e) Apply maximum likelihood for position adjustment(f) **While**
*β*_10_ = *β*_6_
**Do**  
$\beta_{10}=\underset{\forall \;\beta_{9}^{i}}{\text{argmax}}P(\text{x}|\beta_{9})$ **End While****Ensure:**size smoothing(a) Obtain prior probability by using Neyman–Pearson test(b) Obtain null hypothesis, *H*_0_ 
$\begin{array}{cc} \max_{\forall \;\beta^{i}}\;P_{5}(\text{x}|\beta_{i}), & \beta_{i}\in \{\beta_{12}^{a},\ldots ,\beta_{12}^{d}\} \end{array}$(c) Obtain alternative hypothesis, *H*_1_
$\begin{array}{cc} \max_{\forall \;\beta^{j}}\;P_{5}(\text{x}|\beta_{j}), & \beta_{j}\in \{\beta_{11}^{a},\ldots ,\beta_{11}^{d}\} \end{array}$(d) **If**
*H*_1_ is favoured  select 2*^nd^* set of priors**Else**  select 1*^st^* set of priors **End If**(e) For each patch, obtain posterior probability by using Bayes risk(f) Normalize all histograms size for fairer comparison(g) **If**
*P*_5_(*β*_10_|**x**) ≤ *P*_5_(*β_i_*|**x**), 
$\beta_{i}\in \{\beta_{11}^{a},\ldots ,\beta_{11}^{d},\beta_{12}^{a},\ldots ,\beta_{12}^{d}\}$  Size remain constant **Else**  size is updated based on selected side**End If**    (h) Reiterate the process until the patch has converged to a certain size or number of iterationhas exceeded *p* cycles.


We have selected 150 image pairs from various videos from Youtube, which contain challenging scenes between two consecutive frames. Some of the challenges that reduce accuracy and precision of the tracker are illumination changes, shadows, non-rigid object, blur and partial occlusion. The size of the frame varies from 320 × 240 to 960 × 720. The target object is not just a human, but also includes book, animal, ball and many more for both indoor and outdoor environments. However, only one object is tracked each time, since we limit the algorithm to single object tracking. Our tracked object varies in size from frame to frame and from video to video, in which the smallest size is 30 × 6, and the largest size is 341 × 365. Generally, a bigger tracked object will tend to perform better due to the smaller number of candidate patches after patch smoothing. They also tend to overlap with the ground truth after the patch matching process, which later will be fine-tuned by position and size smoothing. For a small size object, the possibility of overlapping is smaller compared with the bigger object, which diminishes the advantage of having position and size smoothing processes. Table 1 shows the parameters used by our algorithm. For both likelihood tests, we found out that 0.0001 gives the best result. In order to get a decent result, it is recommended that *η*_1_ and *η*_2_ should be within [0.00005,0.0005] based on our repeated simulations, while 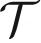
_1_ and 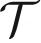
_2_ between 0.6 to 0.75 will give good results. 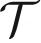
_3_ is used to determine either to continue the test to HSV space or just stop at RGB space, while 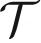
_4_ is the threshold to indicate that the object is already out of the frame. So, correlation value of 0.7 and above will give good results for both tests, where less stringent value will favour RGB space while a more stringent value will favour HSV space. Step size will be determined by 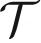
_5_ and we use 0.1 scale of the tracked object's size. Smaller step size will give better accuracy but total number of iterations will be bigger and *vice versa*. 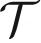
_6_ is used to weight the contribution of maximum correlation and average correlation for size smoothing procedures in deterministic PBOD. We reduced the effect of dependency on maximum correlation alone, as sometimes it may be obtained from noisy or blur patch by adding the averaging components. 0 5 is chosen as it gives good balance between both components. We also analyze the effect of the histogram's size (*N_b_* = 25, 50, 75) where various methods of histogram matching are used for patch matching as shown in Table 2.

The algorithm performance is measured by calculating the Euclidean distance *ε* between the centroid (Ω*_sim_*) of the simulation result and the manually determined ground truth (Ω*_truth_*) of the detected object.

(44) $$ℰ=\sqrt{{\left(\Omega_{\text{sim}}^{x}-\Omega_{\text{truth}}^{x}\right)}^{2}+{\left(\Omega_{\text{sim}}^{y}-\Omega_{\text{truth}}^{y}\right)}^{2}}$$

In terms of average processing speed, the method by Yan *et al.* performs the best with one frame per second (fps), followed by deterministic PBOD, probabilistic PBOD and SIFT-based tracker with 0.27 fps, 0.23 fps and 0.22 fps respectively. The method by Yan *et al.* has the lowest computational burden, since the search scope is limited to the neighbourhood data only, while the other three methods search the whole frames selectively by finding possible keypoints. Computational time for SIFT-based approach is slower because of the complex histogram of gradient and the longer descriptor used for matching as compared with PBOD, which uses simple histogram matching. Table 3 shows the computational limit based on Big-O notation for all four methods.

### 4.1. Histogram Matching

In this subsection, we demonstrated that the most appropriate histogram matching for PBOD is by using Poisson modelling. Partial of the probabilistic PBOD, which is without position and size smoothing is used to validate the best scheme for histogram matching. Five methods have been tested on 150 image pairs for various image conditions, including illumination changes, shadow, non-rigid and homogenous texture object. Those five methods are:

1. Correlation distance, 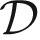
_
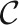
_.
2. Poisson distance, 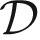
_
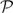
_.
3. Chi square distance, 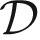
_
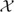
_^2^.
4. Intersection distance, 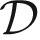
_
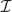
_.
5. Bhattacharyya distance, 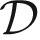
_
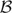
_.

Correlation distance is based on Bradski and Kaehlerv [7] as in Equations (11) and (13), which have been applied in deterministic PBOD. Poisson distance has been implemented in probabilistic PBOD as in Equations (18) and (19), which is based on Zulkifley and Moran [11]. Chi-square distance is taken from Schiele and Crowley [8], where the zero indicates a perfect match. Similar to Section 3.2.1, let *n* and *m* denote previous and current frames' histograms respectively. *m_i_* is the value of *i^th^* bin of the current frame histogram where each histogram is normalized first before matching process. After histogram normalization, the range for both *n_i_* and *m_i_* is [0, 1]. In this paper, we have tested three *N_b_* values, which are 25, 50 and 75.

For a 1-dimensional histogram:

(45) $$\;{𝒟}_{{𝓧}_{1}^{2}}(n,m)=\sum_{i=1}^{N_{b}}\left[\frac{{\left(n_{i}-m_{i}\right)}^{2}}{n_{i}+m_{i}}\right]$$

and for a 3-dimensional histogram:

(46) $${𝒟}_{{𝓧}_{3}^{2}}(n,m)=\sum_{i=1}^{N_{b}}\sum_{j=1}^{N_{b}}\sum_{k=1}^{N_{b}}\left[\frac{{\left(n_{i,j,k}-m_{i,j,k}\right)}^{2}}{n_{i,j,k}+m_{i,j,k}}\right]$$

Intersection distance is based on the work by Swain and Ballard [9] with the output range of [0,1]. It is obtained after normalization process with respect to the total number of pixels in the patch. The method accumulates the minimum bin values between the two histograms as shown in Equations (47) and (48).

For a 1-dimensional histogram:

(47) $${𝒟}_{{ℐ}_{1}}(n,m)=\sum_{i=1}^{N_{b}}\min(n_{i},m_{i})$$

and for a 3-dimensional histogram:

(48) $${𝒟}_{{ℐ}_{3}}(n,m)=\sum_{i=1}^{N_{b}}\sum_{j=1}^{N_{b}}\sum_{k=1}^{N_{b}}\min(n_{i,j,k},m_{i,j,k})$$

Early formulation of Bhattacharyya distance can be traced back to [10], while current implementation is derived from Bradski and Kaehler [7]. The highest distance, one, indicates a total mismatch, while the lowest score, zero, shows the perfect match.

For a 1-dimensional histogram:

(49) $${𝒟}_{{ℬ}_{1}}(n,m)=\sqrt{1-\sum_{i=1}^{N_{b}}\left(\frac{\sqrt{n_{i}.m_{i}}}{\sqrt{\sum_{i=1}^{N_{b}}n_{i}.\sum_{i=1}^{N_{b}}m_{i}}}\right)}$$

and for a 3-dimensional histogram:

(50) $${𝒟}_{{ℬ}_{3}}(n,m)=\sqrt{1-\sum_{i=1}^{N_{b}}\sum_{j=1}^{N_{b}}\sum_{k=1}^{N_{b}}\left(\frac{\sqrt{n_{i,j,k}.m_{i,j,k}}}{\sqrt{\sum_{i=1}^{N_{b}}\sum_{j=1}^{N_{b}}\sum_{k=1}^{N_{b}}n_{i,j,k}.\sum_{i=1}^{N_{b}}\sum_{j=1}^{N_{b}}\sum_{k=1}^{N_{b}}m_{i,j,k}}}\right)}$$

**Figure 11.**
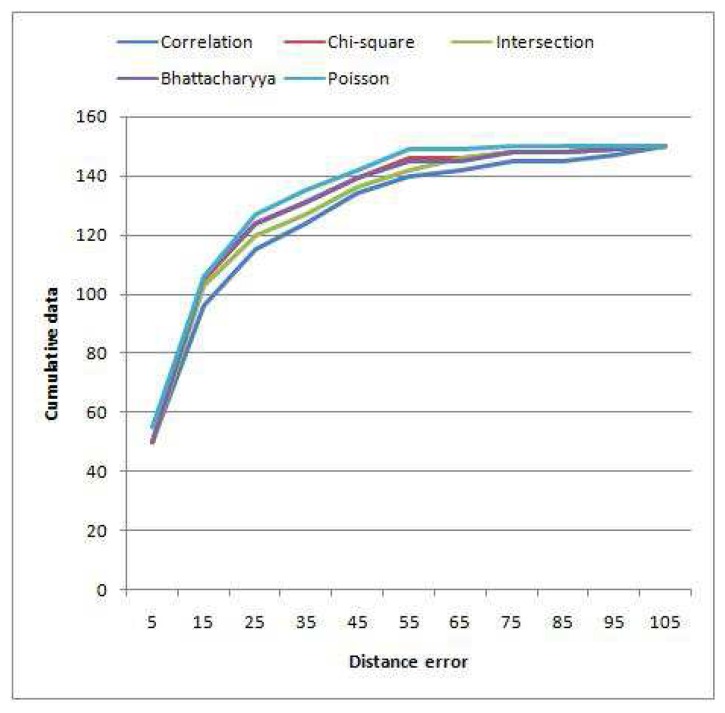
Cumulative distribution of error distance among the histogram matching methods.

Table 4 shows the error distance among the five methods of histogram matching. The centroid of the output patches is used as the reference point to calculate the error. We ran the algorithm for both colour models (RGB and HSV), and the minimum distance is taken since the algorithm for colour space selection is similar for all the methods. The results show that the most suitable histogram matching for PBOD is achieved by using Poisson modelling. Using the Poisson distance, 36.67% of the image pairs achieved an error distance of less than 10 pixels, while the rest just managed to get 33.33%. The most unsuitable method is the correlation distance, which has three wrong detections with error more than 100 pixels. Figure 11 shows the cumulative distribution of the error distance among the matching methods. This again proved that Poisson modelling is the best for PBOD with the steepest curve followed by Bhattacharyya, Chi-square, Intersection and correlation method.

Distance error relative to the patch size 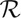
_
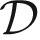
_ is also calculated to show the magnitude of the distance error compared with the object size. The average distance error among the methods is shown in Table 2. We verify the matching test by using three sizes of the histogram, which are 25, 50 and 75 bins. For every bin's size, Poisson test results in the lowest average distance error, while correlation method performs the worst with the highest error for each size. Intersection, chi-square and Bhattacharyya test obtains almost similar error given the same histogram's size. The table also reveals that the error becomes smaller as the total number of bins increases except for the correlation method. The reason is that the distinguishing factor between two similar histograms is widened as the number of bin increases, which leads to more unique feature. Thus, the histogram matching tests have more probabilities of finding the right patch. However, there is no free lunch since the computational time increases as the number of bins increase. From our experience, 50 bins are the most suitable setup given the tradeoff between computational time and accuracy, since the algorithm improves by one percent only for an increment of 25 histogram's bin.

(51) $${ℛ}_{𝒟}=\frac{𝒟}{\max\{\beta_{w}^{\text{new}},\beta_{h}^{\text{new}}\}}\times 100\%$$

### 4.2. Deterministic and Probabilistic PBOD

This subsection is intended to prove that probabilistic PBOD performs better than deterministic PBOD. 120 image pairs are used to verify the performance difference. Again, Equation (44) is used to calculate the error distance of the centroid. Table 5 shows the comparison of error distance between probabilistic and deterministic PBOD. Probabilistic PBOD manages to obtain 48.33% detection with less than 10 pixels error while the deterministic approach manages only 42.50%. Mostly, probabilistic PBOD obtains a better result during illumination changes. Deterministic PBOD uses the hierarchical approach where RGB space is searched first and if the resultant output is within the accepted region, no HSV space will be searched. On the other hand, probabilistic PBOD searches both spaces in parallel, where the best output is selected. If deterministic PBOD obtains a reasonably good result in RGB space, it will not search HSV space where it might obtain better matching. Thus, statistical PBOD manages to obtain better matching in challenging scenes.

**Figure 12.**
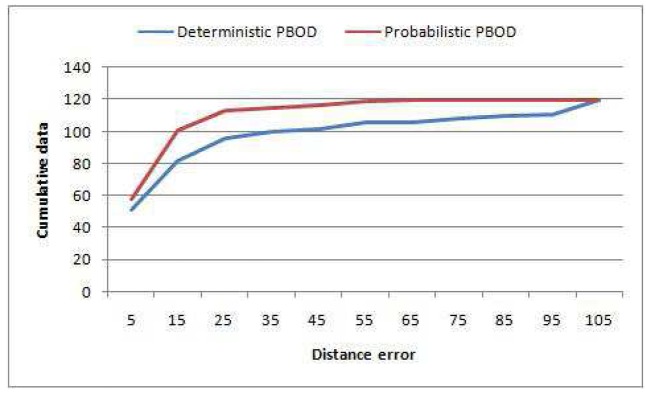
Cumulative distribution of error distance between probabilistic and deterministic PBOD.

**Figure 13.**
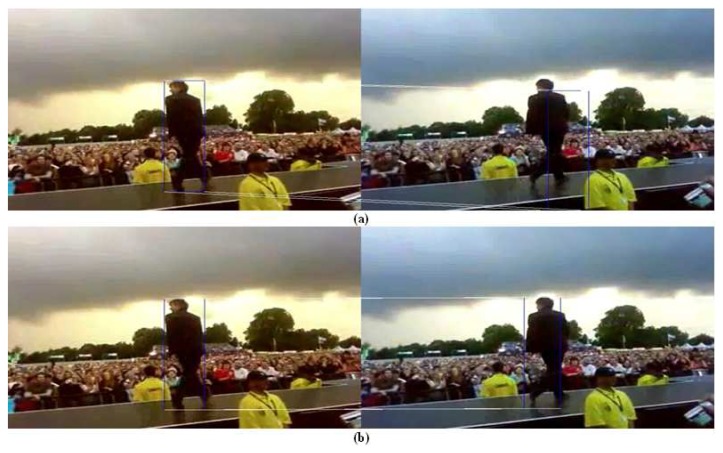
Deterministic and probabilistic PBOD under illumination change: (**a**) Deterministic PBOD (**b**) Probabilistic PBOD.

**Figure 14.**
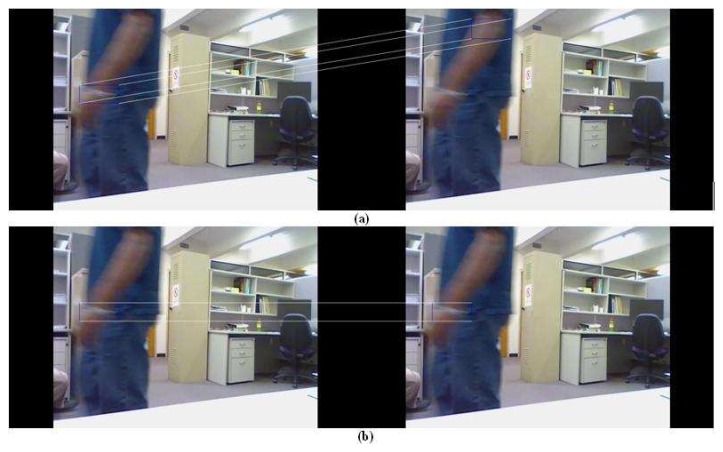
Deterministic and probabilistic PBOD for blur object: (**a**) Deterministic PBOD (**b**) Probabilistic PBOD

Moreover, no error of more than 99 pixels has been observed for the probabilistic PBOD while there are nine image pairs for the deterministic PBOD. The average distance error relative to the output patch size is given in Table 6, which is derived via Equation (51). Figure 12 shows the cumulative distribution of the error between both methods. It shows that 50% of the detections for probabilistic PBOD have less than 11 pixels error distance, while deterministic PBOD requires at least 14 pixels error. Another reason for probabilistic PBOD better performance is due to Poisson modelling where we proved before that it performs the best for PBOD as compared with correlation matching in deterministic PBOD. Poisson matching performs better during a blur case because it does not punish severely neighbourhood shift in histogram's value. The procedures of position and size smoothing of probabilistic PBOD are in the sequential manner where the first step of the patch's direction is very crucial, while probabilistic PBOD calculates the whole search space first before going into any direction. The main downside of searching the whole possible space is longer computational time. Figure 13 is an example of performance difference between probabilistic and deterministic PBOD under sudden illumination change. Figure 14 shows that both PBOD schemes work well in detecting the tracked object even if the object appearance is very blurred.

### 4.3. Probabilistic PBOD, Kernel Tracker and SIFT-based Tracker

**Figure 15.**
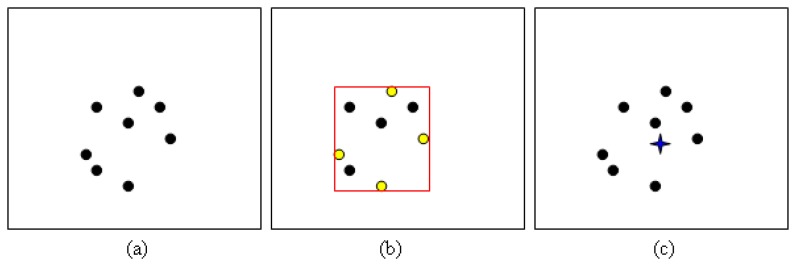
Generating centroid for the SIFT-based tracker (**a**) Matched points of interest (**b**) Constructing a bounding box (**c**) Centroid location is indicated by the blue star.

In this section, we compare probabilistic PBOD with two other trackers, *i.e.*, kernel tracker and SIFT-based tracker. We have tested the algorithms on 120 pairs of video sequences that contain moving objects in various frame sizes. Kernel tracker represents the mean shift approach while SIFT-based tracker represents the feature-based tracker approach. Kernel tracker by Yan *et al.* [18] is chosen as the benchmark due to close similarity to our approach. The method is based on several possible sub-templates, which is built optimally before template voting is performed to select the best match. Their method also performs size smoothing deterministically by varying the template size on the scale of [0.95, 1.05]. On the other hand, SIFT-based tracker is built to compare our algorithm to feature-based tracker by tracking matched point in the consecutive frames. The four corners of PBOD bounding box are used as the reference points for centroid calculation. The centroid for SIFT-based tracker is generated by constructing a bounding box, which uses the extreme points in four directions as shown in Figure 15. Then, the generated bounding box corners are used for the centroid calculation.

**Figure 16.**
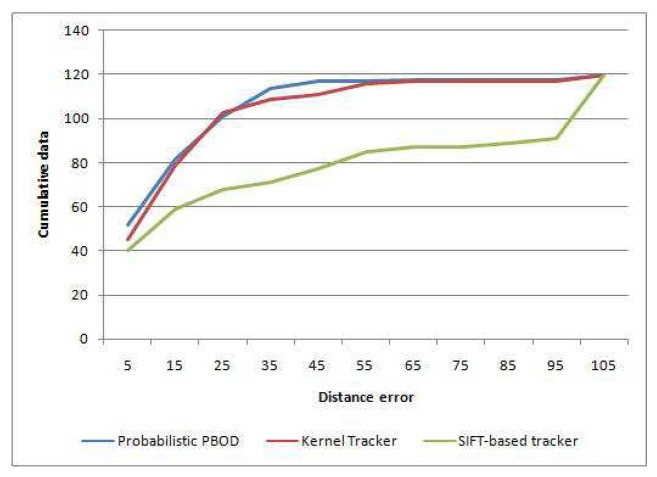
Cumulative distribution of error distance between probabilistic PBOD, Kernel tracker and SIFT-based tracker.

Table 7 shows the distance error analysis among the methods. Probabilistic PBOD performance is the best with 43.3% of the detected object centroid having less than 10 pixels distance from the ground truth centroid. Kernel tracker manages to obtain 37.5% detection with less than 10 pixels distance error while the worst is SIFT-based tracker with just 33.3% detection. Average distance error relative to the patch size (Equation (51)) is given in Table 8, where the error rate of probabilistic PBOD is just 13.95% while kernel tracker has 15.88% and SIFT approach has 35.93%. The cumulative error distance is given in the Figure 16, where 50% of the testbed has less than 13 pixels error distance for the probabilistic PBOD while the kernel and SIFT threshold for 50% accuracy are 24 and 54 pixels respectively.

The main reason for our better performance compared with the method by Yan *et al.* is due to wider search area. Our method built the candidate patches at strategic locations throughout the whole frame, while the sub-templates are built on limited search space. If the object movement is fast, the search area must be wide enough, or else the candidate template or patch will not be generated. Another limitation for the method by Yan *et al.* is that the size variation is assumed to be constant in all directions. The size of the object may increase or decrease in one direction only such as for the case of a human extending his hand, where the size increment will be on that particular hand movement. Our method permits the size smoothing to be performed at certain direction only, which explains better precision. On the other hand, a tracker based on feature detection such as SIFT will not work well if the tracked object has a non-rigid shape. SIFT's signature is built by collecting the neighbourhood data into a histogram of gradient where any background change will heavily affect its value. It also performs poorly if the image is blurred and has shadow noise since the keypoint's descriptor will be different and no match is found. This affects the detection and matching accuracy of the features, which will result in inaccurate observation. Another limitation of SIFT-based tracker is longer computational time, where it is heavily dependent on the number of points detected.

## 5. Conclusions

In this paper, we have shown that probabilistic PBOD works better compared with the deterministic approach in obtaining observation for single object tracking. Probabilistic PBOD registered 48.33% detection with less than 10 pixels error while the deterministic approach only achieved 42.50%. Both PBODs work well in challenging scenes, especially for the problems of low image sharpness, moderate deformation, illumination change, blur, size variation and homogeneous texture, by fusing feature- and template-based approaches. Probabilistic PBOD also performs better than kernel tracker by Yan *et al.* and SIFT-based tracker. The main novelties of probabilistic PBOD are (1) a probabilistic approach to patch-based object recognition, (2) modelling histogram matching by using Poisson and Gaussian distributions, and (3) statistically-based position and size smoothing for better detection accuracy. Robust observation detection allows the algorithm to improve track retention, especially during challenging scenes as the track can still obtain a measurement. This system can be further improved by implementing a stereo vision system as implemented by Marron-Romera *et al.* [38], which results in better detection and tracking accuracy as compared with a single camera system.

## Figures and Tables

**Table 1.** Parameters used by our algorithm.

**Parameter**	**Value**
*η*_1_	0.0001
*η*_2_	0.0001
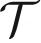 _1_	0.6
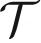 _2_	0.6
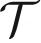 _3_	0.7
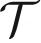 _4_	0.7
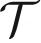 _5_	0.1
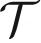 _6_	0.5
*N_b_*	50

**Table 2.** Comparison of the average distance error among histogram matching methods: A: correlation, B: chi-square, C: intersection, D: Bhattacharyya and E: Poisson for various histogram's size.

**Method**	**A**	**B**	**C**	**D**	**E**
** Total no. of histogram's bin:**	**25 bins**				

**Average** 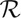 _ 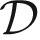 _**(%)**	26.51	22.35	26.88	22.37	19.16
** Total no. of histogram's bin:**	**50 bins**				

**Average** 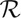 _ 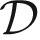 _**(%)**	28.62	19.93	20.87	20.17	16.36
** Total no. of histogram's bin:**	**75 bins**				

**Average** 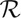 _ 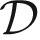 _**(%)**	21.11	17.27	17.84	18.04	15.02

**Table 3.** Big-O notation for the algorithms.

**Method**	**T(n)**
Deterministic PBOD	1 *O*(*n*^3^) + 7 *O*(*n*^2^)
Probabilistic PBOD	2 *O*(*n*^4^)
Yan *et al.*	3 *O*(*n*^2^) + 8 *O*(*n*)
SIFT based approach	*O*((2*n*)^2^) + 2 *O*(*n*^2^)

**Table 4.** Centroid distance for histogram matching methods: A: correlation, B: chi-square, C: intersection, D: Bhattacharyya and E: Poisson.

**Distance Error (Pixel)**	**Number of Image Pairs**				

	**A**	**B**	**C**	**D**	**E**
0–9	50	50	50	50	55
10–19	46	55	53	56	51
20–29	19	19	17	18	21
30–39	9	7	7	7	8
40–49	10	8	9	8	7
50–59	6	7	6	6	7
60–69	2	0	4	0	0
70–79	3	2	2	3	1
80–89	0	0	0	0	0
90–99	2	1	1	1	0
>99	3	1	1	1	0

**Table 5.** Comparison of the centroid distance between deterministic and probabilistic PBOD.

**Distance error (pixel)**	**Number of image pairs**	

	**Deterministic**	**Probabilistic**
0–9	51	58
10–19	31	43
20–29	14	12
30–39	4	2
40–49	2	2
50–59	4	2
60–69	0	1
70–79	2	0
80–89	2	0
90–99	1	0
>99	9	0

**Table 6.** Comparison of the average distance error between deterministic and probabilistic PBOD.

**Method**	**Deterministic PBOD**	**Probabilistic PBOD**
**Average** 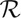 _ 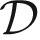 _**(%)**	21.03	10.03

**Table 7.** Comparison of the centroid distance among Probabilistic PBOD, kernel tracker and SIFT-based tracker.

**Distance Error (Pixel)**	**Number of Image Pairs**		

	**Probabilistic PBOD**	**Kernel Tracker**	**SIFT-Based Tracker**
0-9	52	45	40
10-19	30	34	19
20-29	19	24	9
30-39	13	6	3
40-49	3	2	6
50-59	0	5	8
60-69	1	1	2
70-79	0	0	0
80-89	0	0	2
90-99	0	0	2
>99	2	3	30

**Table 8.** Comparison of the average distance error among the PBOD, Kernel Tracker and SIFT-based tracker.

**Methods**	**Probabilistic PBOD**	**Kernel Tracker**	**SIFT-Based Tracker**
Average 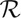 _ 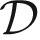 _ (%)	10.03	15.88	35.93
